# Remote Monitoring of Patients With Heart Failure: An Overview of Systematic Reviews

**DOI:** 10.2196/jmir.6571

**Published:** 2017-01-20

**Authors:** Nazli Bashi, Mohanraj Karunanithi, Farhad Fatehi, Hang Ding, Darren Walters

**Affiliations:** ^1^ Australian eHealth Research Centre, CSIRO Brisbane Australia; ^2^ School of Medicine The University of Queensland Brisbane Australia; ^3^ Centre for Online Health The University of Queensland Brisbane Australia; ^4^ School of Allied Medical Sciences Tehran University of Medical Sciences Tehran Islamic Republic Of Iran; ^5^ Department of Cardiology Queensland Health Brisbane Australia

**Keywords:** systematic review, patient monitoring, mobile phone, telemedicine, heart failure

## Abstract

**Background:**

Many systematic reviews exist on the use of remote patient monitoring (RPM) interventions to improve clinical outcomes and psychological well-being of patients with heart failure. However, research is broadly distributed from simple telephone-based to complex technology-based interventions. The scope and focus of such evidence also vary widely, creating challenges for clinicians who seek information on the effect of RPM interventions.

**Objective:**

The aim of this study was to investigate the effects of RPM interventions on the health outcomes of patients with heart failure by synthesizing review-level evidence.

**Methods:**

We searched PubMed, EMBASE, CINAHL (Cumulative Index to Nursing and Allied Health Literature), and the Cochrane Library from 2005 to 2015. We screened reviews based on relevance to RPM interventions using criteria developed for this overview. Independent authors screened, selected, and extracted information from systematic reviews. AMSTAR (Assessment of Multiple Systematic Reviews) was used to assess the methodological quality of individual reviews. We used standardized language to summarize results across reviews and to provide final statements about intervention effectiveness.

**Results:**

A total of 19 systematic reviews met our inclusion criteria. Reviews consisted of RPM with diverse interventions such as telemonitoring, home telehealth, mobile phone–based monitoring, and videoconferencing. All-cause mortality and heart failure mortality were the most frequently reported outcomes, but others such as quality of life, rehospitalization, emergency department visits, and length of stay were also reported. Self-care and knowledge were less commonly identified.

**Conclusions:**

Telemonitoring and home telehealth appear generally effective in reducing heart failure rehospitalization and mortality. Other interventions, including the use of mobile phone–based monitoring and videoconferencing, require further investigation.

## Introduction

### Prior Work

Heart failure is a complex chronic condition that presents debilitating symptoms [1]. There is a high prevalence of heart failure worldwide [2] and, despite advanced medical, pharmacological, and surgical treatment, patient outcomes are poor and hospital readmissions are high [1].

Heart failure clinical outcomes depend largely on how well people self-manage their condition between face-to-face office visits with health care providers. Hence, lack of symptom monitoring and seeking treatment when necessary, particularly between their visits, may result in hospital readmissions in this population. To avoid heart failure exacerbation, patients are encouraged to modify their lifestyle and constantly monitor symptoms related to their condition [1]. Providing patients with the tools to take an active, participatory role in their disease progression and management is important [3].

The high health care costs and poor quality associated with heart failure have led to the development of remote patient monitoring (RPM) systems and cost-effective disease management strategies. RPM uses devices to remotely collect and send data to a health care facility for diagnostic interpretation or monitoring purpose. Such applications might monitor specific vital signs, such as blood pressure, heart rate, or electrocardiogram (ECG), or a variety of indicators for housebound patients. Such systems can be used to facilitate health care by nurses who visit patients at home [4]. RPM comprises a range of noninvasive and patient monitoring approaches that could improve quality of life (QOL) of patients with heart failure who are at high risk of deterioration [5].

The current literature contains research results from numerous trials investigating the clinical, structural, behavioral, or economic effects of RPM interventions on patients with chronic diseases [6]. Recent evidence suggests that RPM component systems have beneficial effects on mortality and hospitalization of patients with heart failure [5]. However, the scope, methods of analysis, results, and quality of systematic reviews are varied and this may cause uncertainty for policy makers, health professionals, and others regarding utilization of the information from existing evidence. Investigating the effect of a wide range of RPM systems on heart failure outcomes is a key aspect in improving such technology-based interventions, but taking it in isolation fails to consider the strength, weakness, and implications for future research.

### Objectives

We undertook this overview to systematically gather, evaluate, and organize the review-level evidence. The aim of this study was to report the highest level of evidence and to identify the RPM intervention that is most effective in improving the clinical outcomes of patients with heart failure. It also aimed to identify existing gaps in this area and to recommend avenues for future research.

## Methods

### Inclusion and Exclusion Criteria

The included records were assessed for eligibility against the study’s inclusion criteria including types of reviews, participants, interventions, and outcomes.

### Types of Reviews

Previous systematic reviews and meta-analyses evaluating the effects of RPM on heart failure and published in peer-reviewed journals or the Cochrane Library were considered eligible for inclusion. Key characteristics of inclusion criteria outlined by the Cochrane Collaboration [7] were used to determine the types of reviews. Depending on the method for analyzing the evidence from primary studies, systematic reviews can be classified as qualitative or narrative reviews and quantitative reviews. We included only quantitative systematic reviews. Conference proceedings, review summaries, editorials, and unpublished studies were excluded.

### Types of Participants

Patients with a diagnosis of heart failure regardless of age, sex, or ethnicity were considered in this review. However, the diagnostic criteria should have been established in the included reviews using standard criteria and New York Heart Association functional classification. Reviews with mixed population were also excluded from this study.

### Types of Interventions

We considered systematic reviews and meta-analyses that investigated the effectiveness of RPM interventions for patients with heart failure. These interventions applied information and communication technology (ICT) for mentoring, supporting physical or mental health, and/or monitoring of any vital signs, biometric and/or data related disease (signs and symptoms) from patients to health care providers. The systematic reviews that only investigated the effect of telediagnosis were excluded. We also excluded structured telephone support from this overview because the definition of RPM used in this overview considered structured telephone support distinctly different from RPM interventions.

### Types of Outcomes

We sought data for outcomes in the following categories:

- Patient-oriented outcomes, such as knowledge and self-care, health status, and well-being
- Health service–oriented outcomes, which include rehospitalization, emergency department visits, and length of stay

Reviews were included if the primary or secondary outcomes from included studies were related to the clinical or behavioral effects of RPM on patients with heart failure. Systematic reviews and meta-analyses that investigated only the cost, feasibility, or uptake of RPM systems were excluded.

### Search Methods for Identification of Studies

A comprehensive and systematic search was performed using the electronic sources PubMed, EMBASE, CINAHL (Cumulative Index to Nursing and Allied Health Literature), and the Cochrane Library from 2005 to 2015. A sensitive search strategy was developed and refined by an experienced medical information specialist. A combination of MeSH (Medical Subject Headings) terms as well as key terms related to telemedicine, heart failure, and systematic reviews were used to search PubMed for all relevant studies. This search strategy was modified for searching the other databases according to their user guide. Details of the search strategy are presented in Multimedia Appendix 1.

### Data Collection and Analysis

#### Selection Procedure

**Figure 1:**
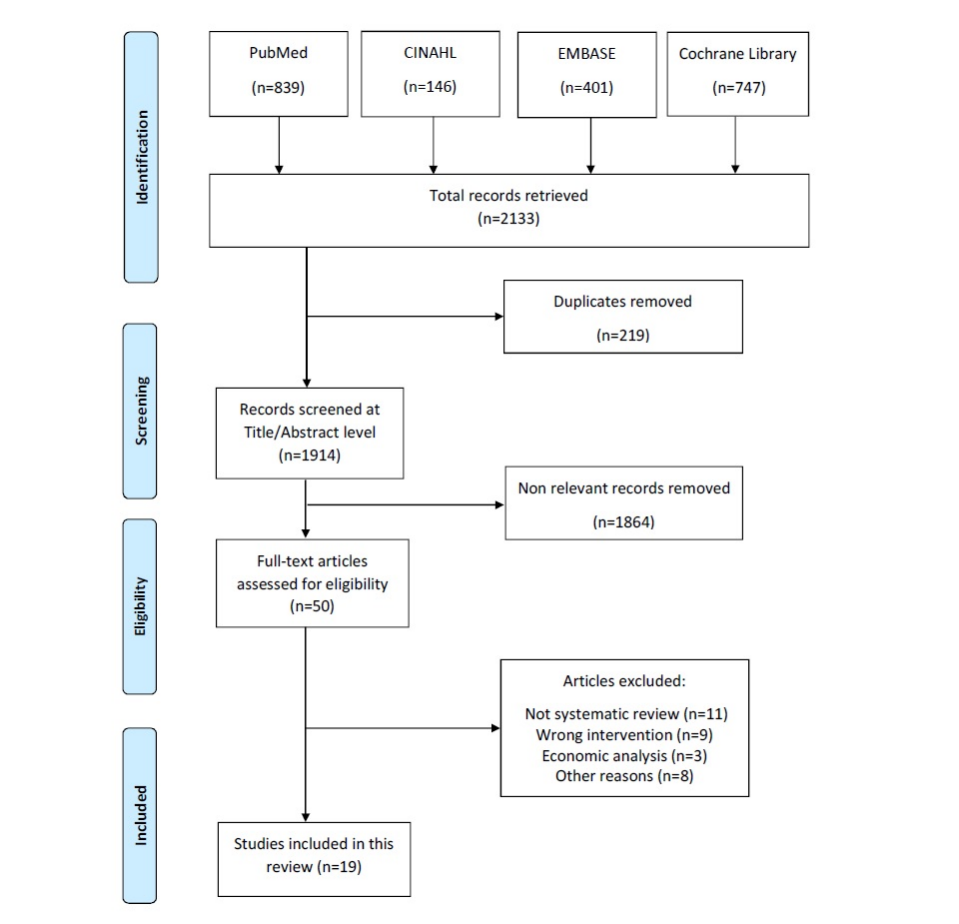
Study flow diagram.

Studies reviewing telemonitoring, telehealth, and remote monitoring outcomes in patients with heart failure were selected by 2 independent reviewers. Records that did not clearly meet the inclusion criteria were excluded. Studies were excluded if they investigated the effects of RPM on patients with mix of chronic diseases. As shown in Figure 1, our initial search resulted in 2133 records. The reviewers read all titles and abstracts to remove duplicate studies (219). On the basis of the inclusion criteria, nonrelevant records were excluded (1864). If there was any discrepancy, the reviewers discussed the issues and reached a consensus. Because of resource limitation, reviews published in languages other than English were excluded from the analysis. A number of records (11) were excluded as they were not systematic reviews, and 20 systematic reviews were excluded because of the following reasons: wrong interventions, economic analysis, or other reasons. This yielded a final number of 19 systematic reviews included in this study.

#### Summaries of Reviewed Systematic Reviews

To allow consistent reporting of results across reviews, we extracted and descriptively summarized each study’s results using standardized language. Then we summarized and narratively reported results of the reviews to enable identification of broad conclusions within and across reviews. A table was developed to extract key characteristics of each review (Table 1). The information sought included general details related to systematic reviews (eg, authors and year of publication) and more specific details about the types of interventions, outcome variables, sample (number of included studies in each review), results, and methodological shortcomings.

### Methodological Quality of Reviewed Studies

The methodological quality of included systematic reviews and meta-analyses was examined by using the Assessment of Multiple Systematic Reviews (AMSTAR) tool [27]. The tool is a validated instrument and assesses the degree to which review methods avoided bias by evaluating the methods against 11 distinct criteria. The process of scoring was performed by independent assessors. Each AMSTAR item was rated as yes (clearly done), no (clearly not done), can’t answer, or not applicable, based on the published review report. A review that adequately met all of the 11 criteria was considered to be a review of the highest quality. The quality rating was as follows: AMSTAR score (out of 11 criteria) rating 8 to 11 as high quality, 4 to 7 as moderate quality, and 3 or lower as low quality.

As reported by the authors of this overview, systematic reviews included studies that ranged from high (well-designed and well-conducted studies) to low quality (studies with serious methodological limitations; Table 2). A small number of reviews were highly selective about the quality of the studies they included; for example, 3 systematic reviews [9,17,21] specified that only randomized controlled trials (RCTs) were eligible for inclusion. However, these measures did not ensure that included studies were of high quality.

#### Question 1: A Priori Design

Only 3 reviews [9,11,18] included in this study established inclusion criteria and a priori design before commencing with the literature search, data collection, and abstraction. The rest of the systematic reviews did not frame their research inclusion criteria and lacked a priori design.

#### Question 2: Duplicate Study Selection and Data Extraction

Of 19 reviews, in 10 reviews [8-11,13,14,16-18,20] the process of screening was performed by 2 independent authors. In 8 reviews the authors did not clarify this process [12,15,19,22-26].

#### Question 3: Comprehensive Search

The analysis of this question, which required at least two electronic sources and one supplementary strategy in order to be scored as comprehensive search, showed that all reviews used at least two electronic databases to search primary studies. The frequently used databases were MEDLINE, CINAHL, and EMBASE. Although all reviews reported the years and databases searched, only 12 reviews had comprehensive literature review based on the AMSTAR criteria [8,9,11,12,14,16,17,19-22,25].

#### Question 4: Inclusion of Gray Literature

Among 19 reviews included in this overview, only 5 reviews reported searching for gray literature regardless of their publication type [8,9,11,21,25]. Most reviews focused on primary studies that were published in English-language journals.

#### Question 5: Included and Excluded Studies Provided

Most reviews presented a list of included studies. However, only 2 reviews [19,22] reported a list of excluded studies in their article or as a supplementary material.

#### Question 6: Characteristics of the Included Studies

Characteristics of the original studies with respect to the participants, intervention, and outcomes were presented in 15 reviews in the form of a table or illustration [8-11,13,15,16,18-25]. A range of characteristics from primary studies such as the mean age of patients, duration of follow-up, and severity of disease was reported in the reviews.

#### Question 7: Quality Assessment of the Primary Studies

The methodological quality or risk of bias of primary studies included in the reviews was appraised in 10 reviews [8,9,11,12,14,18,20-22,25] out of the 19 reviews. These 10 systematic reviews provided their methods to assess included studies either using a quality scale (such as checklist with composite scores) or predefined risk of bias criteria. Furthermore, the quality of individual studies was reported in a meaningful format of a grade or score by these reviews.

#### Question 8: Scientific Quality of Included Studies Used Appropriately in Formulating Conclusions

Out of the 19 reviews, 7 reviews [8,9,11,14,21,22,25] formally assessed the scientific rigor of the primary studies and integrated the results of the methodological quality into the final conclusions and made recommendations for future studies.

#### Question 9: Appropriateness of Methods Used to Combine Studies’ Findings

There were 7 reviews [8-10,13,18,19,21] that used a specific method such as chi-square test to combine the results.

#### Question 10: Publication Bias

The risk of publication bias was reported in 2 reviews [9,13]. As shown in Table 2, the rest of the systematic reviews did not assess publication bias.

**Table 1:** Characteristics of the included systematic reviews.

Author	Population (mean age, disease severity)	Type of study^a^	Intervention (length of follow-up)	Outcome variables	No. of studies (sample size)	Results	Methodological shortcomings
Kotb et al [8]	10,193 patients (mean age 44-80 years, NYHA^b^ class I-IV, most II-III)	30 RCTs^c^	Telemonitoring, structured telephone support, video monitoring (6-26 months)	HF^d^ mortality, all-cause hospitalization, HF hospitalization	30 (10,193)	Reduced mortality and HF hospitalization in telemonitoring and STS.	Not reported.
Inglis et al [9]	Mean age 57-78 years, NYHA class I-IV, most II-IV	25 RCTs and 5 abstracts	Telemonitoring and structured telephone support (3-15 months)	All-cause mortality, hospitalization (all-cause, HF), cost, QOL^e^, and LOS^f^	30	Telemonitoring reduced all-cause mortality. Both telemonitoring and STS reduced HF hospitalizations, cost, and improved QOL.	Not reported.
Nakamura et al [10]	3337 patients (mean age 65 years, NYHA class I-IV)	13 RCTs	RPM^g^ including PDAs and mobile phones	Mortality, medication management	13 (3337)	RPM significantly reduced the risk of mortality.	Types of control groups were varied among reviewed studies. Patients’ medications were different among studies.
Pandor et al [11]	6561 patients, 1918 patients recently discharged (mean age 57-78 years, NYHA class I-IV, most II-IV)	20 RCTs	RPM including telemonitoring and structured telephone support (3-12 months, recently discharged patients; 6-22 months, patients with stable HF)	All-cause mortality, hospitalization (HF, all-cause), QOL, system acceptability, and LOS	20	Reduction in mortality and all-cause hospitalization in recently discharged patients, improvement in QOL.	Reviewed studies were heterogeneous in terms of monitored parameters, HF selection criteria, sample size, and follow-up duration.
Smith [12]		20 (RCTs and observational studies)	Telemonitoring and structured telephone support	Readmission to hospital for any reason	20	HF readmission reduced but evidence for all-cause readmission is inconclusive.	Studies were heterogeneous.
Xiang et al [13]	7530 patients (mean age 69 years, NYHA class I-IV, most II-IV)	33 RCTs	Telemonitoring (6-26 months)	All-cause mortality, HF hospitalization, HF-related LOS	33 (7530)	Significant reduction in all-cause mortality, HF hospitalization, HF-related LOS.	In some studies, sample was small and underpowered to detect a significant association.
Ciere et al [14]	Not reported (mean age 61-78 years, mild or moderate class of HF)	12 (11 RCTs and 1 pre-post study)	Telehealth (6-12 months)	Knowledge, efficacy, and self-care	12	Associations between telehealth and knowledge, and telehealth and self-care were mixed. TH had no effect on self-efficacy.	Limited number of studies, poor methodological quality, and mixed findings.
Radhakrishnan and Jacelon [15]	20-214	14 (12 RCTs, 8 pre-post designs, 2 quasi-experimental, and 1 pilot control)	Telehealth (1-12 months)	Self-management	14	Some level of improvement in self-care.	Studies had small sample size or low power for statistical analyses. There was a risk of recall bias.
Giamouzis et al [16]	57-710 (mean age 44-86 years, NYHA class I-IV)	12 RCTs, 2 multinational	Telemonitoring (6-26 months)	All-cause mortality, all-cause rehospitalization, cardiovascular hospitalization, ED^h^ visits, bed days, days lost due to death	12 (57-710)	Mixed results.	Some studies had small sample size and, therefore, were underpowered to detect significant associations.
Clarke et al [17]	3480 (mean age range 55-85 years, NYHA class I-IV)	13 RCTs	Telemonitoring (3-15 months)	All-cause mortality, all-cause emergency hospital admission, LOS	13 (3480)	Overall reduction in all-cause mortality and HF hospital admission, no significant effects were found in all-cause emergency and hospital admission, LOS, medication adherence, or cost.	Small sample sizes, diverse control groups, interventions, and approaches in interpreting data and contacting patients.
Polisena et al [18]	3082 patients (mean age 52-75 years, NYHA class I-IV)	17 (8 RCTs and 9 observational studies)	Telemonitoring (1-12 months)	Mortality (all-cause, HF, or cardiovascular), hospitalization (HF, all-cause), ED visits (HF, all-cause), primary care or specialist visits, and home visits	17 (3082)	The number of ED visits, all-cause hospitalizations, and mortality reduced in telemonitoring group. Results related to the number of primary care or specialist visits and home visits were inconclusive.	Diverse patient population and length of follow-up, lack of proper blinding and randomization, and the wide range of home telemonitoring interventions.
Klersy et al [19]	8612 (age range 54-81 years, NYHA III-IV)	32 (20 RCTs, 12 cohort studies)	RPM (3-18 months)	Mortality, hospitalizations (all-cause, HF)	32 (8612)	The rate of mortality, hospitalizations for any cause, and hospitalizations for HF in both RCTs and cohort studies were reduced.	Not reported
Chaudhry et al [20]	Mean age 67.7 years, NYHA I-IV	9 RCTs (2 single-site and 7 multicenter)	Telephone or automated symptom monitoring	All-cause mortality, hospitalizations (all-cause, HF), event rate, and ED visits	9	Results were mixed. Telephone-based monitoring was less expensive.	High-quality trials regarding the effectiveness of automated forms of telemonitoring are scarce.
Clark et al [21]	4264 (mean age range 57-75 years, NYHA II-IV)	14 RCTs (not reported)	Telemonitoring or structured telephone support (3-16 months)	Mortality (all-cause), readmission (all-cause, HF), QOL, cost, adherence, patient acceptability	14 (4264)	QOL improved and all-cause mortality reduced. No significant effect was found on all-cause readmission and HF readmission.	Small number of trials, short-term follow-up.
Dang et al [22]	Mean age range 53.2-79 years, NYHA II-IV	9 RCTs (not reported)	Home telehealth remote monitoring (3-12 months)	All-cause mortality, readmissions (all-cause, HF), ED visits, LOS, clinic visit (scheduled, unscheduled)	9	The impact of telemonitoring on health care utilization, mortality, and cost is positive. The results for other outcome variables were mixed.	Interventions were varied in terms of technology, duration, and the process of data analysis. The patient populations were heterogeneous in terms of NYHA class, HF duration, and socioeconomic status.
Hughes and Granger [23]	Mean age 63.75 years	4 RCTs, pre-post survey	Technology-based intervention to promote self-management (30 days to 12 months)	Self-management, rehospitalization, satisfaction, QOL, and cost	4 (733)	Technology-based interventions resulted in improved outcomes related to self-management, rehospitalizations, costs, and QOL.	The number and quality of the studies are low.
Maric et al [24]	3184 (NYHA I-IV)	RCT, pre-post survey	Telemonitoring interventions (1-18 months)	Hospitalization, emergency room costs, QOL, bed days, home visits, combined events (hospital admissions, ED access/visits, mortality, left ventricular ejection fraction, and psychological moods	56	The reviewed studies showed a general trend toward improvement of outcome measures such as QOL, self-efficacy, hospitalization, and ED visits.	The majority of studies were not randomized and many had small sample sizes.
Martinez et al [25]	Mean age range 48-83 years, NYHA I-IV	RCT, descriptive, noncontrolled clinical series	Home telemonitoring (3-24 months)	Mortality, feasibility, readmissions, QOL, LOS, and cost	42	Many studies showed reduction in mortality, hospital readmissions, and length of hospital days and improved QOL.	Not reported.
Schmidt et al [26]	Not reported	19 RCTs	Telemonitoring	Mortality and rehospitalization, QOL, health-economic benefits, acceptance of home monitoring by patients, acceptance by clinicians and influence on doctor-patient relationship, significance of telemonitoring for patient compliance	19	The available scientific data on vital signs monitoring are limited, yet there is evidence for a positive effect on some clinical end points, particularly mortality. Nonetheless, any possible improvement in patient-reported outcomes, such as QOL, still remains to be demonstrated.	Not provided.

^a^Type of study: RCT, cohort study, or case study and multicenter or single-center study.

^b^NYHA: New York Heart Association.

^c^RCT: randomized controlled trial.

^d^HF: heart failure.

^e^QOL: quality of life.

^f^LOS: length of stay.

^g^RPM: remote patient monitoring.

^h^ED: emergency department.

^i^STS: structured telephone support

#### Question 11: Conflicts of Interest

Interestingly, 10 reviews [9-11,13,16,18,21,22,24,25] provided information related to the project’s source of funding and conflict of interest. Assessing the methodological quality of included systematic reviews and meta-analyses revealed that the quality of 12 systematic reviews [8,10,11,13,14,16,18-22,25] was moderate as they were scored between 4 and 7. A total of 6 systematic reviews [12,15,17,23,24,26] had a very low quality (scored 3 and less) and only 1 systematic review was identified as highest quality (scored 10) [9]. Overall, many of the reviews displayed important limitations. For example, only 3 systematic reviews [9,11,18] had referred to their a priori design, such as a published protocol, and 2 systematic reviews [9,13] assessed the likelihood of publication bias of reviewed studies.

**Table 2:** Methodological quality of systematic reviews based on AMSTAR (Assessment of Multiple Systematic Reviews) scores.

Author	Q1^a^	Q2	Q3	Q4	Q5	Q6	Q7	Q8	Q9	Q10	Q11	Total
Chaudhry et al [20]	No	Yes	Yes	No	No	Yes	Yes	No	No	No	No	4
Ciere et al [14]	No	Yes	Yes	CA^b^	No	CA	Yes	Yes	No	Yes	No	5
Clarke et al [17]	No	Yes	Yes	No	CA	No	No	No	No	No	No	2
Clark et al [21]	No	Yes	Yes	Yes	CA	Yes	No	No	Yes	No	Yes	6
Dang et al [22]	No	CA	Yes	No	Yes	Yes	Yes	Yes	No	No	Yes	6
Giamouzis et al [16]	No	Yes	Yes	No	No	Yes	No	No	No	CA	Yes	4
Hughes and Granger [23]	No	No	No	No	No	Yes	No	No	No	No	CA	1
Inglis et al [9]	Yes	Yes	Yes	Yes	No	Yes	Yes	Yes	Yes	Yes	Yes	10
Klersy et al [19]	No	No	Yes	No	Yes	Yes	No	No	Yes	No	No	4
Kotb et al [8]	No	Yes	Yes	Yes	CA	Yes	Yes	Yes	Yes	No	No	7
Maric et al [24]	No	No	No	No	No	Yes	No	No	No	No	Yes	2
Martinez et al [25]	No	No	Yes	Yes	No	Yes	Yes	Yes	CA	No	Yes	6
Nakamura et al [10]	CA	Yes	No	No	No	Yes	No	No	Yes	No	Yes	4
Pandor et al [11]	Yes	No	Yes	Yes	No	Yes	Yes	Yes	No	No	Yes	7
Polisena et al [18]	Yes	Yes	No	No	No	Yes	Yes	No	Yes	No	Yes	6
Radhakrishnan and Jacelon [15]	No	No	No	CA	No	Yes	No	No	No	No	No	1
Schmidt et al [26]	No	No	No	No	No	No	No	No	No	No	No	0
Smith [12]	No	No	Yes	No	No	No	Yes	No	No	No	No	2
Xiang et al [13]	No	Yes	No	No	No	Yes	CA	No	Yes	Yes	No	4

^a^Q: question.

^b^CA: can’t answer.

**Table 3:** Taxonomy of interventions and examples of outcomes reported.

Intervention	Example of outcome
Telemonitoring	Mortality, hospitalization (all-cause, HF^a^), QOL^b^, length of stay, emergency department visits
Home telehealth	Mortality, hospitalization (all-cause, HF), QOL, self-care, knowledge
Mobile phone	Self-management, QOL, cost
Video monitoring	Mortality, HF hospitalization
Personal digital assistant devices	Mortality

^a^HF: heart failure.

^b^QOL: quality of life.

### Synthesis of Results and Rating the Evidence of Effectiveness

We applied 4 steps in developing the data integration tables based on the study by Ryan et al [28]. These were first identifying, extracting, and summarizing relevant information from each review for a comprehensive picture of the characteristics of the systematic review. Second, results for the outcomes of the review were assessed and translated to standardized statements. Third, results including RPM interventions and heart failure outcomes were mapped onto a taxonomy (Table 3) using language specific to the field of RPM interventions. Fourth, the implications of the review were assessed after the mapping step clarified the level of evidence available for each intervention. These steps were taken to assist with synthesizing and rating the evidence across systematic reviews with complex and diverse interventions [28].

## Results

### Types of Interventions

This overview appraised and summarized 19 systematic reviews that consisted of a broad range of RPM interventions such as telemonitoring (telemonitoring includes the collection and transmission of clinical data between a patient at a distant location and a health care provider through a remote interface so that the provider may conduct a clinical review of such data or provide a response relating to such data) [29], home telehealth (home telehealth comprises remote health care delivery or monitoring between a health care professional and a patient outside of clinical settings, in their home or assisted living residence) [29], and integration of electronic transfer of physiological data via mobile phones, wearable electronic devices, or implantable electronic devices. Table 1 provides a summary of the reviews’ characteristics that focused on RPM in heart failure outcomes. Studies were published from 2006 to 2015 and consisted of a minimum of 4 [23] and a maximum of 56 original studies [24] with the methodology of RCT, cohort studies, and/or pre-post studies. The follow-up period ranged from 1 to 26 months.

Table 4 shows that 13 systematic reviews investigated the effect of telemonitoring and home telehealth. Among these, 4 reviews [9,11,12,21] investigated the effect of structured telephone support but as mentioned earlier, the results related to structured telephone support excluded from this review. One systematic review included 3 RCTs that examined the effect of videoconferencing and compared the intervention with usual care or telephone support [8]. One RCT and 1 pre-post study cited by 2 systematic reviews [10,23] examined mobile phone interventions. In 1 systematic review [10], 11 RCTs investigated the effect of PDAs. The devices used in those RCTs were widely varied. A total of 4 systematic reviews investigated the effect of home telehealth on heart failure clinical outcomes.

**Table 4:** Interventions’ effectiveness.

Intervention category	Types of interventions	Examples of interventions	Reviews mapped to this category	Statements of effectiveness
Telemonitoring	14 SRs^a^ examined the effect of telemedicine including telemonitoring and home telehealth. Among these, there were 4 reviews that also investigated the effect of structured telephone support.	Telephone-based symptom monitoring, automated monitoring of signs and symptoms, automated physiological monitoring (such as body weight, heart rate, arterial blood pressure, ECG^b^ recordings), and other data.	[8,9,11-13,16-21,23,24,26]	There is sufficient evidence that telemonitoring interventions have an effect on clinical outcomes of HF^c^ including a reduction in mortality, HF hospitalization, and all-cause hospitalization and improvement in QOL^d^.
Video monitoring	One SR covering 3 RCTs^e^ that implemented videoconferencing as main intervention and compared it with usual care or telephone support.	Monitoring patients’ body weight, blood pressure, heart rate, and/or ECG. Some systems also included consultations.	[8]	There is not enough evidence to support conclusions about the effect of video monitoring on HF outcomes as the number of trials is small.
Mobile phone monitoring	Two SRs including 1 RCT and 1 pre-post study examined mobile phone–based interventions.	Monitoring body weight, blood pressure, heart rate, or ECG. Patient consultation.	[10,23]	Based on this review, there is insufficient evidence to determine the effect of mobile phone–based monitoring on HF clinical outcomes.
PDA devices	One SR of 11 RCTs investigated the effect of PDA devices. The devices used in those RCTs were varied.	Monitoring body weight, blood pressure, heart rate, or ECG. Patient consultation.	[10]	There is some evidence that the use of PDA devices is effective in reducing HF mortality. There is not enough evidence to make decisions about the effect of PDA interventions on the other clinical outcomes of HF.
Home telehealth	Four SRs investigated the effect of home telehealth on the clinical outcomes of HF.	Monitoring vital signs and/or ECG, individualized education, medication reminder.	[14,15,22,25]	Based on the results of this review there is some level of evidence from trials that home telehealth has an effect on HF clinical outcomes such as mortality, health care utilization, and QOL.

^a^SR: systematic review.

^b^ECG: electrocardiogram.

^c^HF: heart failure.

^d^QOL: quality of life.

^e^RCT: randomized controlled trial.

### Population

Among 19 reviews, 16 reviews [8-11,13,14,16-25] reported the mean or range of participants’ age and/or New York Heart Association heart failure classification. The highest reported mean age was 86 years [16] and the lowest was 44 years [16]. Of the reviews, 10 systematic reviews [8-11,13,16-18,20,25] documented the New York Heart Association classes I-IV, 2 systematic reviews reported classes II-IV [21,22], 1 systematic review reported classes III-IV [19], and 1 systematic review described participants as having a mild or moderate class of heart failure [14]. The rest of the reviews did not report heart failure classification (Table 1). One systematic review investigated studies focused on patients with heart failure following discharge after a recent episode of hospitalization [11].

### Effect of Interventions or Clinical Outcomes

Statements related to intervention effectiveness were determined using the evidence rating scheme for each review and summarized within each intervention category (Table 4). Out of 19 systematic reviews, 9 reviews showed a reduction in all-cause mortality [8-11,13,16,17,20,21] and 5 reviews showed a reduction in all-cause hospitalizations [11,18,19,23,25]. A total of 6 reviews reported a reduction in heart failure hospitalization [8,11,17,20-22], 2 reviews reported a reduction in length of hospital stay [9,11], and 1 systematic review reported a reduction in emergency department visits [18]. Improvement of QOL was reported in 3 reviews [11,21,23] and self-care in 1 review [15].

Telemonitoring has been shown to reduce mortality (risk ratio, RR, 0.66, 95% CI 0.54-0.81, *P*<.001) [9]. Also, 24 hours telemonitorirng over 7 days (hazard ratio 0.76, 95% CI 0.49-1.18), and telemonitoring during office hours (hazard ratio 0.62, 95% credible interval 0.42-0.89) [11], (risk ratio 0.77, 95% CI= 0.61-0.97, *P* ≥.01 ) [21], (odds ratio 0.53,CI 0.36-0.80) [8] has reduced the rate of HF mortality. The significant effect of telemonitoring on health care utilization was also reported in several systematic reviews (risk ratio 0.72, 95% CI 0.61-0.85) [13], (odds ratio 0.64, 95% CI 0.39-0.95) [8], (risk ratio 0.79, 95% CI 0.67-0.94, *P>*.001) [21], (RR 0.93, 95% CI 0.87-0.99, *P* ≥.01) [19].

## Discussion

### Principal Findings

This overview reports evidence from 19 unique systematic reviews that have synthesized trials and other studies evaluating the effects of RPM interventions on heart failure outcomes. Information on a wide range of outcomes was sought. The most commonly measured and reported outcomes were mortality [9,11,17,20,21] and heart failure rehospitalization [9,17-20], but many others (Table 5) reported and helped to inform RPM outcomes [15-17,19]. Limitations in the quality of the systematic reviews included in this overview (Table 2) demonstrate that there is a lack of high-quality evidence of RPM interventions. This could be due to the fact that several of the articles were not designed and structured as systematic reviews based on validated assessment tools such as AMSTAR.

The results of this overview demonstrate that telemonitoring has beneficial effects on clinical outcomes of heart failure, including a reduction in mortality, heart failure hospitalization, and all-cause hospitalization and an improvement in QOL [8,9,12,14,16-18,20,21,24]. It can be concluded that key elements of telemonitoring including physiological monitoring of blood pressure, heart rate, weight, and ECG must form an integral part of the routine care of patients with heart failure.

Although the number and quality of systematic reviews that examined the impact of home telehealth interventions on heart failure outcomes were limited, the collected evidence suggests that home telehealth interventions have positive effects of reduced health care utilization [25] and improved QOL [15]. However, these interventions do not appear to have any effect on knowledge and self-care [19].

Despite recent advances in telecommunications technology that have facilitated clinical use of videoconferencing, the results of this overview (Table 4) suggest that there is a lack of evidence to support the effectiveness of mobile phone–based monitoring and video monitoring. This may be due to the limited number of studies investigating the effects of these interventions on patients with heart failure [8,13,23]. Although mobile phone–based monitoring and videoconferencing have not shown to be as effective as telemonitoring in improving heart failure outcomes, these are accessible, convenient, and widely acceptable to patients [30,31]. Furthermore, mobile phones have the capacity to assist patients to receive feedback from and communicate with health care professionals. With the high penetration of mobile phones in most countries and rapid advancement of the wireless network, these interventions have the potential to be incorporated into highly interactive ICT-based platforms to deliver health care and disease self-management programs [31].

This overview has a number of strengths including a comprehensive search strategy, duplicate screening, data extraction, and the use of a validated instrument (AMSTAR) to assess the methodological quality of included reviews. Mapping the evidence using the validated assessment tool and synthesizing the results of included systematic reviews, rather than reporting results of individual primary studies, helped us to differentiate between outcomes where there was sufficient evidence related to the heart failure RPM interventions and identify the gap in the evidence.

There are a number of limitations that must be kept in mind when interpreting the results of this overview. This overview only reported articles published in English. In addition, the information related to the RPM interventions and their outcomes has not been retrieved from primary studies; therefore, the results of this overview are limited by the data reported in the systematic reviews. Furthermore, all systematic reviews are prone to publication bias and, therefore, such bias may have been transferred to our overview. There is also a possibility that individual studies were included in more than one systematic review; therefore, double counting is inevitable and this may affect the results [32].

Considering the lack of high-quality reviews in the current literature, we recommend that more robust methods are utilized in conducting and reporting systematic reviews. This will lead to less evidence but higher quality and, therefore, result in a well-organized field of literature that is more interpretable by researchers.

**Table 5:** Clinical outcomes reported by the systematic reviews.

Author	Clinical outcome^a^											
	1	2	3	4	5	6	7	8	9	10	11	12
Kotb et al [8]		Yes	Yes	Yes								
Inglis et al [9]	Yes			Yes		Yes				Yes		Yes
Nakamura et al [10]	Yes								Yes			
Pandor et al [11]	Yes		Yes	Yes		Yes				Yes		
Smith [12]			Yes								Yes	
Xiang et al [13]	Yes			Yes						Yes		
Ciere et al [14]							Yes	Yes				
Radhakrishnan and Jacelon [15]								Yes				
Giamouzis et al [16]	Yes		Yes	Yes	Yes					Yes		
Clarke et al [17]	Yes				Yes				Yes	Yes		Yes
Polisena et al [18]	Yes	Yes	Yes	Yes	Yes							
Klersy et al [19]	Yes		Yes	Yes								
Maric et al [24]												
Chaudhry et al [20]	Yes		Yes	Yes								
Clark et al [21]	Yes		Yes	Yes		Yes						Yes
Dang et al [22]	Yes		Yes	Yes	Yes	Yes				Yes	Yes	Yes
Hughes and Granger [23]						Yes					Yes	Yes
Martinez et al [25]	Yes					Yes				Yes		Yes
Schmidt et al [26]	Yes		Yes			Yes						

^a^Clinical outcomes: 1, all-cause mortality; 2, heart failure mortality; 3, all-cause hospitalizations; 4, heart failure–related hospitalizations; 5, emergency department visits; 6, quality of life; 7, knowledge; 8, self-care; 9, medication adherence or medication management; 10, length of stay; 11, readmission; 12, costs.

### Implications for Practice

The overview of systematic reviews demonstrates telemonitoring to be effective in reducing mortality and rehospitalization of patients with heart failure [8,9,12,14,16-18,20,21,24]. This required key elements of telemonitoring such as monitoring of blood pressure, heart rate, weight, and ECG. Health care professionals who seek a more rigorous and stronger RPM intervention that is evidenced to improve clinical outcomes of patients with heart failure may adopt telemedicine key elements. Additionally, the intervention taxonomy may assist health care providers to identify a range of interventions available in relation to specific outcomes.

### Implications for Research

Despite the evidence of effectiveness that resulted from the studies included in this overview, many areas of uncertainty remain, and interventions using mobile phone and video monitoring require further rigorous assessment [8,10,23]. Rapid advances and the ubiquitous availability of mobile phones have created new perspectives on ICT-based health care delivery systems [31]. Although the current evidence is not sufficient to support the effect of mobile phone and video monitoring on heart failure mortality or health care utilization, it is evident that their uptake and adherence is high [30,31]. This is because these interventions can be delivered anywhere at any time and for extended periods and consequently facilitate regular communication and behavioral maintenance. Lack of sufficient information in the current evidence indicates a clear need for further high-quality research on mobile phone–based and videoconferencing interventions. Hence, we recommend further investigation of the effects of these interventions in future. There is also a need to determine the intensity and duration of telemonitoring and home telehealth interventions.

## Appendix

Multimedia Appendix 1 Search strategy.

## Abbreviations

AMSTAR: Assessment of Multiple Systematic Reviews
CINAHL: Cumulative Index to Nursing and Allied Health Literature
ECG: electrocardiogram
ICT: information and communication technology
QOL: quality of life
RCT: randomized controlled trial
RPM: remote patient monitoring
