# Supplementary material for: Remote Monitoring of Patients With Heart Failure: An Overview of Systematic Reviews
Source: J Med Internet Res. 2017 Jan 20;19(1):e18. doi: 10.2196/jmir.6571 (PMC5291866; doi:10.2196/jmir.6571)
Supplement: Multimedia Appendix 1 [file jmir_v19i1e18_app1.pdf]

Systematic review: Key words

PubMed Search Strategy: (2005 - Date)

Heart Failure:
"Heart Failure"[Mesh] OR Heart Failure OR Heart Failures OR Cardiac Failure OR Cardiac Failures OR Myocardial Failure OR Myocardial Failures OR Heart Decompensation OR Cardio-Renal Syndrome OR Cardio-Renal Syndromes OR Cardiorenal Syndrome OR Cardiorenal Syndromes OR Renocardiac Syndrome OR Renocardiac Syndromes OR Reno-Cardiac Syndrome OR Reno Cardiac Syndrome OR Reno Cardiac Syndromes OR Reno-Cardiac Syndromes OR Paroxysmal Dyspnea OR Paroxysmal Dyspneas OR Cardiac Edema OR Cardiac Edemas OR Cardiac Aedema

Review:
"systematic review" OR review OR overview

"review"[Publication Type] OR "review literature as topic"[MeSH Terms] OR "systematic review"[All Fields] OR "meta-analysis"[Publication Type] OR "meta-analysis as topic"[MeSH Terms] OR "meta-analysis"[All Fields]

OR

"meta-analysis"[Publication Type] OR "meta-analysis as topic"[MeSH Terms] OR "meta-analysis"[All Fields]

Cochrane:

Telemedicine or "Mobile Health" or mHealth or mHealths or Telehealth or Telecare or Tele-care or "Tele Care" or Telehomecare or "Tele-homecare" or Telenursing or Telepractice or "Tele-practic"e or eHealth or "Remote Consultatio"n or "Remote Consultations" or "Teleconsultation" or Teleconsultations or "Remote consultation" or "Remote Consultations" or "Remote Care" or "Remote Monitoring" or Telemetry or Telemetries or "Remote Sensing Technology" or "Remote Sensing Technologies" or Videoconference or Videoconferencing or "Video Conference" or "Video Conferencing" or "Video consultation" or "Video Consultations" or "Video Visit" or "Video Visits" or "Video Call" or Software or "Computer Program" or Computer-Assisted or "Computer mediated" or Computerized or Computerised or "Decision Support System" or "Cellular Phone" or "Cellular Phones" or "Cellular Telephone" or "Cellular Telephones" or "Cell Phone" or "Cell Phones" or "Transportable Cellular Phone" or "Transportable Cellular Phones" or Smartphone or Smartphones or "Smart Phones" or "Smart Phone" or "Mobile Phone" or "Mobile Phones" or "Mobile Telephone" or "Mobile Telephones" or "Car Phone" or "Car Phones" or "Portable Cellular Phone" or "Portable Cellular Phones" or "Mobile application" or "Mobile Applications" or "Mobile App" or "Mobile Apps" or SMS or "Text message" or "Text Messaging" or Android or iOS or iPhone or "App Store" or "Google Play" or Skype or Facetime or Microcomputers or "Tablet computer" or "Tablet Computers" or "Handheld Computer" or "Handheld Computers" or "Personal Digital Assistant" or Internet or Online or Web based or "Web-based" or Website or Websites or "Web Site" or "Web Sites" or Webcam or "Electronic Mail" or "Electronic Mails" or Email or Emails or Telecommunication or Telecommunications or "Computer Networks" or "Computer Networks" or "Computer Networking" or "Communication Networks" or Modem or Wireless or Bluetooth or "Virtual Clinic" or "Virtual Clinics" or "Virtual Visit" or "Virtual Visits" or "Virtual Reality" or "Cyber Space"

EMBASE

1. telemedicine:ti :ab
2. home care services:ti :ab
3. monitoring, ambulatory:ti :ab
4. monitoring, physiologic:ti :ab
5. tele med:ti :ab
6. tele-med:ti:ab
7. telecare: ti:ab
8. telemonitor: ti:ab
9. tele-monitor:ti:ab
10. teleconsult:ti:ab
11. telecommunicate:ti:ab
12. telehealth:ti:ab
13. telemetry.ti:ab
14. tele-consult:ti:ab
15. tele-health:ti:ab
16. telehome:ti:ab
17. tele-home:ti:ab
18. telehomecare:ti:ab
19. tele-homecare:ti:ab

20. telematic:ti:ab
21. telenurs:ti:ab
22. tele-nurs:ti:ab
23. remote consult:ti:ab
24. remote monitoring:ti:ab
25. systematic review/ or systematic review :ti:ab
28. meta-analysis:ab
29. intervention: ti:ab
30. ehealth :ti:ab
31. e-health :ti:ab

CINAHL (EBSCOhost)

1. (MH "Telecommunications") or (MH "Interactive Voice Response Systems") or (MH "Telehealth") or (MH "Telemedicine") or (MH "Remote Consultation") or (MH "Telenursing")
2. TX (tele med*)
3. TX (tele-med*)
4. TX (telemed*)
5. TX (telecare*)
6. TX (telemonitor*)
7. TX (tele-monitor*)
8. TX (teleconsult*)
9. TX (telecommunicat*)
10. TX (telehealth*)
11. TX (telemetry)
12. TX (tele-consult*)
13. TX (tele-health*)
14. TX (telehome)
15. TX (tele-home)
16. TX (telehomecare)
17. TX (tele-homecare)
18. TX (telematic)
19. TX (telenurs*)
20. TX (tele-nurs*)
21. TX (remote consultation)
22. TX (remote monitoring)
23. TX (ehealth)
24. TX (e-health)
25. ((MH "Systematic Review")) OR (TX (systematic review))
26. (MH "Meta Analysis")
27. TI (intervention*)

Cochrane Library

1. MeSH descriptor: [Telemedicine] explode all trees
2. MeSH descriptor: [Home Care Services] this term only
3. MeSH descriptor: [Monitoring, Ambulatory] this term only
4. MeSH descriptor: [Monitoring, Physiologic] this term only

5. tele-med*
6. tele med
7. telecare*
8. telemonitor*
9. tele-monitor*
10. teleconsult*
11. telecommunicat*
12. telehealth*
13. telemetry
14. tele-consult*
15. tele-health*
16. telehome
17. tele-home
18. telehomecare
19. tele-homecare
20. telematic
21. telenurs*
22. tele-nurs*
23. remote consult*
24. remote monitoring
25. e-health
26. ehealth
27. ehealth*
28. e-health*
29. mhealth
30. mhealth*
31. m-health
32. m-health*
32. systematic review OR meta-analysis

Heart Failure

1. heart failure[tw]
2. ventricular dysfunction,
3. left"[MeSH Terms:noexp]
4. cardiomyopathy[tw]
5. left ventricular ejection fraction[tw])
6. medline[sb])
7. Heart Failure[tiab]
8. left ventricular dysfucntion[tiab]
9. cardiomyopathy[tiab]
10. cardiac resynchronization[tiab]
11. cardiac failure[tiab]
12. left ventricular systolic dysfunction[tiab]
13. LV dysfunction[tiab]

14. left ventricular diastolic dysfunction[tiab]
